# Reappraisal writing relieves social anxiety and may be accompanied by changes in frontal alpha asymmetry

**DOI:** 10.3389/fpsyg.2015.01604

**Published:** 2015-10-21

**Authors:** Fen Wang, Changming Wang, Qin Yin, Kui Wang, Dongdong Li, Mengchai Mao, Chaozhe Zhu, Yuxia Huang

**Affiliations:** ^1^State Key Laboratory of Cognitive Neuroscience and Learning and IDG/McGovern Institute for Brain Research, Beijing Normal UniversityBeijing, China; ^2^Center for Collaboration and Innovation in Brain and Learning Sciences, Beijing Normal UniversityBeijing, China; ^3^Beijing Anding Hospital, Capital Medical UniversityBeijing, China; ^4^Beijing Key Laboratory of Mental DisordersBeijing, China; ^5^Beijing Institute for Brain DisordersBeijing, China; ^6^Key Laboratory of Mental Health, Institute of Psychology, Chinese Academy of SciencesBeijing, China

**Keywords:** expressive writing, reappraisal, emotion regulation, EEG, mental health

## Abstract

It is widely reported that expressive writing can improve mental and physical health. However, to date, the neural correlates of expressive writing have not been reported. The current study examined the neural electrical correlates of expressive writing in a reappraisal approach. Three groups of participants were required to give a public speech. Before speaking, the reappraisal writing group was asked to write about the current stressful task in a reappraisal manner. The irrelevant writing group was asked to write about their weekly plan, and the non-writing group did not write anything. It was found that following the experimental writing manipulation, both reappraisal and irrelevant writing conditions decreased self-reported anxiety levels. But when re-exposed to the stressful situation, participants in the irrelevant writing group showed increased anxiety levels, while anxiety levels remained lower in the reappraisal group. During the experimental writing manipulation period, participants in the reappraisal group had lower frontal alpha asymmetry scores than those in the irrelevant writing group. However, following re-exposure to stress, participants in the reappraisal group showed higher frontal alpha asymmetry scores than those in the irrelevant writing group. Self-reported anxiety and frontal alpha asymmetry of the non-writing condition did not change significantly across these different stages. It is noteworthy that expressive writing in a reappraisal style seems not to be a fast-acting treatment but may instead take effect in the long run.

## Introduction

“Expressive writing” is also known as “writing emotional disclosure,” “writing emotional expression,” and “therapeutic writing,” etc. Expressive writing is a way for people to disclose and express their feelings and thoughts about their previous experiences or coming events, and can be beneficial for both physical and mental health (Pennebaker and Chung, 2011). Pennebaker and Beall (1986) were the first to experimentally examine its effects. They asked participants to write about personally traumatic life events, and the results showed that writing about both the emotions and the facts surrounding a traumatic event decreased health center visits in the 6 months after the experiment. Since the work of Pennebaker, expressive writing has been widely studied, and meta-analysis suggest that it has positive effects on health (Frattaroli, 2006). In healthy individuals, writing about stressful experiences reduced health care utilization (Harris, 2006). In clinical populations, meta-analysis (Frisina et al., 2004, 2005) of expressive writing studies also found a significant benefit for health. Among the benefits of expressive writing, an improvement in emotional status is noteworthy. Some researchers (Lepore and Smyth, 2002) even think that an improvement in emotional regulation is the key link for health promotion.

In contrast to the large body of behavioral and peripheral physiological studies, studies of the neural correlates of expressive writing are surprisingly scarce. The current development of neural scientific techniques has provided the possibility of studying the neural basis of psychological activities and regulations non-invasively. For example, the electroencephalography/event-related potential (EEG/ERP) technique can show the temporal course of the cognitive reappraisal of emotional stimuli (Thiruchselvam et al., 2011; Blechert et al., 2012; Parvaz et al., 2012), while brain regions involved in reappraisal (Drabant et al., 2009; Kanske et al., 2011; McRae et al., 2012) have been revealed by functional magnetic resonance imaging (fMRI). The current study therefore planned to explore the neural electrical correlates of expressive writing with EEG, since this experimental approach permits the use of ecologically-valid behavioral tasks, such as that used here.

Extensive literature has revealed that frontal EEG asymmetry is related to emotion-related traits and states (Coan and Allen, 2004). In particular, Davidson (1993) has proposed that frontal EEG asymmetry may reflect the brain activities that moderate or mediate approach and withdrawal tendencies in responding to emotional stimuli. Greater left than right frontal cortical activity is associated with “approach” motivational processes, which can be positive (e.g., enthusiasm) or negative (e.g., anger) in affective valence. In contrast, greater right as compared to left frontal activity is associated with “withdrawal” motivational processes (Harmon-Jones et al., 2010). Alpha power is most typically examined, and is regarded as an index of the inverse of cortical activity. Frontal alpha asymmetry (FAA) is typically calculated by subtracting the natural log of left hemisphere alpha power from the natural log of right hemisphere alpha power: ln(right alpha)–ln(left alpha). It is reported that a relatively right-sided resting frontal EEG asymmetry may be associated with anxiety (for a review, see Thibodeau et al., 2006) and may predict poorer performance in emotional regulation tasks (e.g., Hannesdóttir et al., 2010). Among individuals assigned to stressful situations (specifically, a social rejection situation), greater left relative to right prefrontal intracortical activity at baseline was associated with more self-reported approach-oriented emotions and more adaptive cardiovascular profiles (Koslov et al., 2011). Decreases in anxiety levels elicited by certain treatments (e.g., Petruzzello and Landers, 1994; Moscovitch et al., 2011) are accompanied by a shift in FAA from greater relative right activity to greater left activity. The current study uses FAA as an index of the neural electrical changes induced by expressive writing.

Compared to its known beneficial effects, the mechanisms underlying expressive writing are largely unknown and, in particular, lack empirical evidence. Researchers have proposed that expressive writing may take effect through several, possibly complementary, mechanisms, such as exposure, habituation, cognitive reconstruction/reappraisal and benefit pursuing (King and Miner, 2000; Kloss and Lisman, 2002; Guastella and Dadds, 2008; Pennebaker and Chung, 2011). Exposure requires participants to describe the upsetting experiences in detail, thus leading to a disinhibition effect. Repeated exposure can help people habituate to aversive emotions. Cognitive reconstruction induces people to change false beliefs and regard the stressful situations in some other way. In benefit finding, people may find out positive elements in traumatic or stressful events. The underlying mechanisms and neural correlates of these different approaches may differ from one another. To avoid the confounding of these different methods of regulation, the current study focused on cognitive reappraisal as the research target.

As a frequently-used emotional regulation method, reappraisal has been reported to improve the subjective experience, lighten the mental burden of, and reduce the peripheral physiological responses elicited by stressful events (e.g., Boden et al., 2012; Finkel et al., 2013; Gruber et al., 2014). ERP (e.g., Hajcak and Nieuwenhuis, 2006; MacNamara et al., 2011; Blechert et al., 2012), EEG (e.g., Parvaz et al., 2012) and fMRI (e.g., Goldin et al., 2008; Vanderhasselt et al., 2013; Winecoff et al., 2013; Allard and Kensinger, 2014) studies have also shown corresponding effects of reappraisal at the neural level. In these studies, reappraisal was conducted through a thinking method. As mentioned in the theories of expressive writing (Lyubomirsky et al., 2006; Pennebaker and Chung, 2011), the writing may help to release mental stress, order thoughts, improve introspection, and decrease profitless emotional rumination. For these aspects, expressive writing may have unique advantages relative to the thinking method. Thus, the effect of reappraisal writing, and relevant neural correlates, were explored in the present study.

This study used an evaluated speaking task (e.g., Hofmann et al., 2009) to elicit anxiety in participants. Reappraisal writing was used to alleviate their anxious emotions. Reappraisal writing was compared to irrelevant writing and non-writing conditions. Self-reported emotional state and EEG signals were recorded throughout the experiment. The aim of the current study was to examine the effects of reappraisal writing on anxiety alleviation, and the corresponding neural electrical changes.

## Materials and methods

### Participants

A total of 92 university students participated as paid volunteers. Data from 12 participants were excluded from further processing and analysis for failure to follow the task requirements (*n* = 3), equipment malfunction (*n* = 4), and excessive EEG artifacts (*n* = 5). The final 80 participants belonged to three groups: the reappraisal writing group (20–26 years of age, mean [*M*] = 22.86, standard deviation [*SD*] = 1.78, 15 male, 13 female), the irrelevant writing group (19–27 year of age, *M* = 22.96, *SD* = 2.01, 13 male, 14 female), and the non-writing group (19–28 years of age, *M* = 23.12, *SD* = 2.15, 13 male, 12 female). All of the participants were right-handed, as assessed with the Handedness Questionnaire (Li, 1983), and had normal or corrected-to-normal vision. They reported no history of neurological or mental health problems. Written informed consent was obtained from all of the participants. This study was approved by the research ethics committee of the School of Brain and Cognitive Sciences in Beijing Normal University. All study procedures were conducted in accordance with the current version of the Declaration of Helsinki.

### Materials

**Figure 1:**
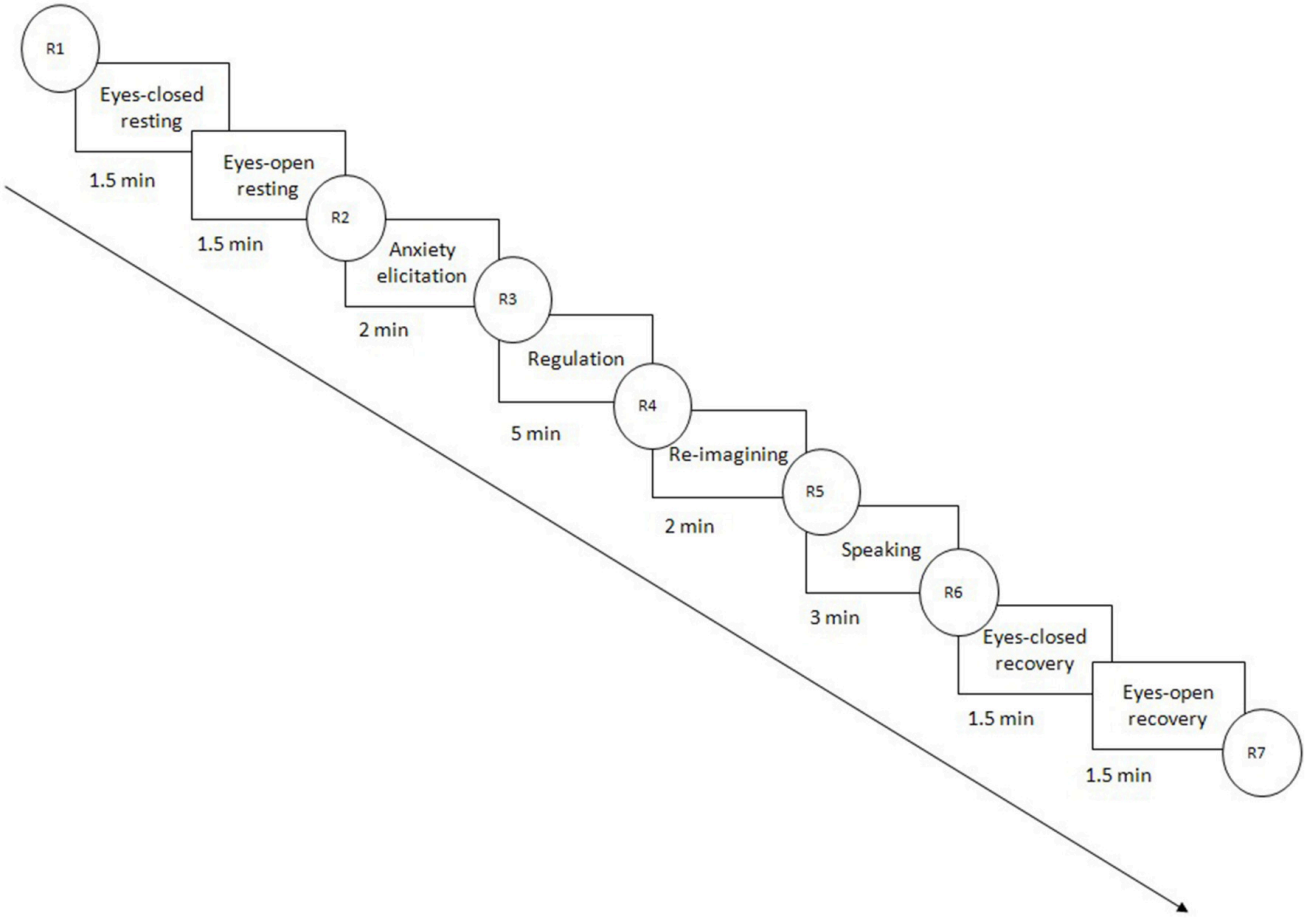
**A schematic diagram of the experimental procedures**. R1 to R7 represent self-reported ratings of anxiety level.

The Personal Report of Confidence as a Speaker (PRCS) (Wang et al., 1999), the State-Trait Anxiety Inventory (STAI-T) (Spielberger et al., 1983; Wang et al., 1999), the Behavioral Inhibition/Activation System Scale (BIS/BAS) (Carver and White, 1994; Li et al., 2008), and the Emotion Regulation Questionnaire Chinese Revised Version (ERQ-CRV) (Wang et al., 2007) were used to examine whether the participants in the three groups differed in levels of anxiety trait, behavioral inhibition/activation or emotional expression. At the very beginning of the main study, participants were asked to report their anxious feeling levels on a 10-point Likert scale (see Procedure and R1 in Figure 1).

### Procedure

On attending the laboratory, the main experimenter (a female) introduced participants briefly to the experiment. Then, participants were asked to complete the questionnaires and inventories detailed above before beginning the main part of the study, during which EEG signals were recorded. Figure 1 shows the procedures of the main study.

The first and the second steps were eyes-closed and eyes-open resting periods, respectively, and each lasted 1.5 min. The third period was anxiety elicitation, during which the participants (all were native Chinese speakers) were told that they would give an impromptu English speech at the end of the experiment (for instruction details, see Supplementary Material). This evaluated speaking task is evidenced to be able to evoke anxiety successfully in laboratory circumstances (e.g., Hofmann et al., 2009). After being informed of the speech task, they were instructed to imagine the speech scenarios or recall previous embarrassing experiences in a speech situation. The imagining or recalling lasted for 2 min. The fourth period was regulation. The reappraisal writing group was asked to regulate their emotions by writing in a reappraisal style. The participants in the irrelevant writing group were asked to regulate their emotions by writing things irrelevant to the speech task. The participants of the non-writing group did not do a writing task and were asked to just sit still. The regulation period lasted for 5 min. Materials written by participants were checked by the main experimenter before further data processing. Data from participants who did not write texts as instructed were excluded from further analysis. The fifth period was re-imagining. The participants were asked to imagine the speech scenario again. The re-imagining instructions in this period were the same as those in the third period. The sixth period was speaking. In this period, the participants were asked to give a 3-min English speech in front of three experimenters (the main experimenter and two assistants, two females and one male) and a camera that had been turned on. The seventh and the eighth periods were the same as the first and the second periods, which were eyes-closed and eyes-open recovery periods, respectively, each lasting 1.5 min.

The participants were asked “how anxious are you now?” at seven experimental time points (see R1 to R7 in Figure 1). R1 was followed immediately by the eyes-closed resting stage. R2 to R7 were conducted immediately after each previous task stage. They were asked to rate their anxiety level on a 10-point visual analog Likert scale (“0” = not anxious at all, “1–3” = mildly anxious, “4–5” = moderately anxious, “6–8” = very anxious, “9” = extremely anxious). The instructions were presented, and self-report ratings were collected, via E-Prime 2.0. EEG data were collected simultaneously throughout the entire experiment.

### EEG data collection, reduction and analysis

EEG data were recorded with a 128-channel Geodesic Sensor Net, using the Electrical Geodesic Instrument system (Tucker, 1993). The input impedance of the amplifier was 200 kΩ. The electrodes were placed in an extended 10–20 International system and referenced to Cz during recording. Offline, all EEG activity was re-referenced to a global average reference. The horizontal electro-occulogram (EOG) was recorded with two electrodes placed at the outer canthi of both eyes. The vertical EOG was recorded with electrodes on the infra-orbital and supra-orbital regions of both eyes. All impedances were kept below 50 kΩ. The EEG from each electrode site was digitized at 500 Hz and filtered with a band-pass filter with a range of 0.01–200 Hz.

Raw EEG data were then down-sampled to 250 Hz and band-pass filtered between 0.5 and 35 Hz. Independent components analysis was used to identify and correct EOG artifacts. Following EOG artifact removal, EEG data were visually screened for motion-related, and other artifacts. If an artifact occurred in any one channel, data from all channels were removed for those periods. The rest of data were concatenated together for subsequent processing. All of the above offline analyses were performed using EEGLAB 9.0.4.4b, a Matlab-based open-source toolbox (Delorme and Makeig, 2004).

EEG data from three homologous pairs of frontal electrodes of the International 10–20 system (i.e., F3 and F4 at the mid-frontal region, F7 and F8 at the lateral-frontal region, and FP1 and FP2 at the pre-frontal region) were selected for subsequent processing. These electrode positions are frequently used sites in the measures of frontal alpha asymmetry (e.g., Coan and Allen, 2003; Thibodeau et al., 2006; Stewart et al., 2008; Gatzke-Kopp et al., 2014; Meyer et al., 2014). To obtain an overall measure for frontal asymmetry, right-side and left-side electrodes were pooled into a pair of frontal right and frontal left electrodes (Frontal R-L). The task stages included in subsequent processing were the eyes-open resting stage, the anxiety elicitation stage, the regulation stage, the re-imagining stage, the speaking stage and the eyes-open recovery stage. At each site of interest, a 1-min EEG segment in the middle of the remaining artifact-removed data of each task stage was selected for subsequent processing. EEG segments were subsequently segmented into bins of 10 s each with a 50% overlap. A continuous wavelet transformation was used to estimate the spectral power density (μV2/Hz) in the alpha (8–12 Hz) band. The average alpha power density values for each stage at each site were then transformed using a natural log function. A measure of EEG hemispheric asymmetry was then derived (ln[right alpha]–ln[left alpha]) for each participant at each electrode. Therefore, an increase in FAA reflected increased activation in the left-side frontal cortex, and a decrease meant increased right-side activation.

### Statistics

The behavioral and EEG data were analyzed for variables of interest using the statistical package for the social sciences (SPSS v. 20.0).

## Results

### Measures of personality

Nine One-way ANOVAs were used to test whether the three groups had significant differences in PRCS, STAI-T, BIS/BAS, ERQ or R1 scores. Participants in the three groups were well-matched for levels of speech confidence, trait anxiety, behavioral inhibition/activation tendency, emotion expression, and anxious state: there were no significant group differences in PRCS scores [*F*_(2, 77)_ = 0.184, *P* > 0.05], STAI-T scores [*F*_(2, 77)_ = 1.195, *P* > 0.05], BIS/BAS scores [BIS scores, *F*_(2, 77)_ = 0.224, *P* > 0.05; BAS Driving, *F*_(2, 77)_ = 0.210, *P* > 0.05; BAS Fun Seeking, *F*_(2, 77)_ = 0.079, *P* > 0.05; BAS Reward Responsiveness, *F*_(2, 77)_ = 1.422, *P* > 0.05; BAS scores, *F*_(2, 77)_ = 0.246, *P* > 0.05]; ERQ scores [*F*_(2, 77)_ = 2.149, *P* > 0.05], or R1 score [*F*_(2, 77)_ = 0.398, *P* > 0.05].

### Self-reported anxiety

**Figure 2:**
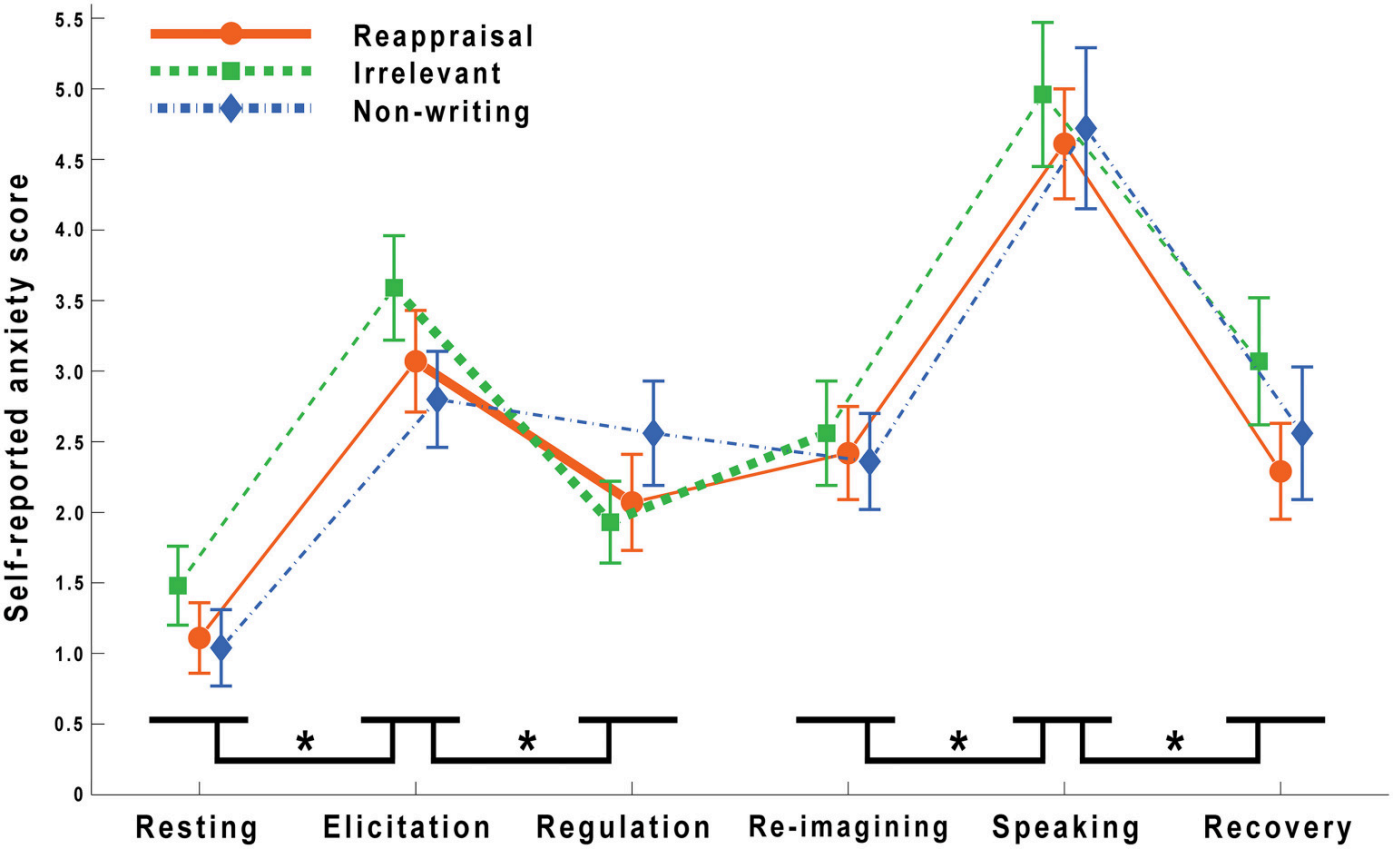
**Trends of self-reported anxiety levels (M ± SEM) throughout the entire task**. The asterisks at the bottom of the figure indicate significant differences of the means of three groups in adjacent stages (^*^P < 0.001). There were significant interactions between group and stage during the elicitation, regulation, and re-imagining stages. The thicker line segments indicate significant differences in the following simple effect analyses (elicitation vs. regulation in the reappraisal condition, *P* < 0.001; elicitation vs. regulation in the irrelevant condition, *P* < 0.001; regulation vs. re-imagining in the irrelevant condition, *P* < 0.01).

Self-reported anxiety scores at the end of each task stage (R2 to R7) are listed in Table 1, and trends of anxiety levels throughout the entire task are shown in Figure 2.

**Table 1:** **Self-reported anxiety scores (M ± SD) of each group in each task stage**.

**Group**	**Resting**	**Elicitation**	**Regulation**	**Re-imagining**	**Speaking**	**Recovery**
Reappraisal (*n* = 28)	1.11 ± 1.34	3.07 ± 1.88	2.07 ± 1.78	2.42 ± 1.77	4.61 ± 2.04	2.29 ± 1.82
Irrelevant (*n* = 27)	1.48 ± 1.45	3.59 ± 1.93	1.93 ± 1.49	2.56 ± 1.91	4.96 ± 2.67	3.07 ± 2.35
Non-writing (*n* = 25)	1.04 ± 1.34	2.80 ± 1.71	2.56 ± 1.83	2.36 ± 1.68	4.72 ± 2.87	2.56 ± 2.35

First a three (group: reappraisal writing, irrelevant writing and non-writing) by six (stage: resting, elicitation, regulation, re-imagining, speaking, and recovery) repeated measures ANOVA was carried out. The results showed a significant main effect of stage [*F*_(5, 77)_ = 69.430, *P* < 0.001]. There was no significant group main effect [*F*_(2, 77)_ = 0.349, *P* > 0.05], nor an interaction between group and stage [*F*_(10, 77)_ = 1.125, *P* > 0.05]. Multiple comparisons with Šidák correction indicated that the elicitation evoked higher anxiety levels than the resting stage (*P* < 0.001). After the regulation period, the anxiety levels were lower than those after the elicitation stage (*P* < 0.001). The speaking stage caused higher anxiety than the re-imagining (*P* < 0.001). After the recovery, participants' self-reported anxiety dropped significantly (*P* < 0.001) relative to the speaking stage. There was no difference found between the regulation stage and the re-imagining stage after Šidák correction, *P* > 0.05 (*P* < 0.05 if with least significant difference).

From Figure 2 it can be seen that there might be some interactions between group and stage during the elicitation, regulation, and re-imagining periods. Thus, two three (group: reappraisal writing, irrelevant writing and non-writing) by two (stage: elicitation and regulation, or regulation and re-imagining, respectively) analyses were used to test these effects. Significant interactions between group and stage (elicitation and regulation) were revealed [*F*_(2, 77)_ = 7.137, *P* < 0.01]. Simple effects analysis revealed that reappraisal decreased the anxiety level significantly [*F*_(1, 27)_ = 15.12, *P* < 0.001] and irrelevant writing also decreased the anxiety level significantly [*F*_(1, 26)_ = 40.51, *P* < 0.001] while this effect was not significant in the non-writing group, [*F*_(1, 24)_ = 0.78, *P* > 0.05]. There was also a significant interaction between group and stage for the comparison of regulation and re-imagining [*F*_(2, 77)_ = 3.672, *P* < 0.05]. When re-exposed to the anxious situation after reappraisal, participants' anxiety level did not change significantly [*F*_(1, 27)_ = 2.84, *P* > 0.05]. However, in the irrelevant writing group, participants' anxiety scores increased significantly [*F*_(1, 26)_ = 8.52, *P* < 0.01]. There was no change found in the non-writing group [*F*_(1, 24)_ = 0.80, *P* > 0.05].

### Frontal alpha asymmetry

The means and standard deviations of FAA for each electrode pair and each group are listed in Table 2.

**Table 2:** **Alpha asymmetries (M ± SD) for each electrode pair and group**.

**Group**	**Electrode pair**	**Resting**	**Elicitation**	**Regulation**	**Re-imagining**	**Speaking**	**Recovery**
Reappraisal (*n* = 28)	FP2-FP1	0.06 ± 0.20	−0.02 ± 0.21	−0.06 ± 0.23	0.05 ± 0.22	−0.01 ± 0.28	0.07 ± 0.20
	F4-F3	0.01 ± 0.23	−0.07 ± 0.24	−0.12 ± 0.20	0.00 ± 0.23	−0.07 ± 0.19	0.02 ± 0.23
	F8-F7	−0.07 ± 0.20	−0.15 ± 0.22	−0.21 ± 0.18	−0.07 ± 0.20	−0.13 ± 0.19	−0.06 ± 0.18
	Frontal R-L	−0.00 ± 0.11	−0.08 ± 0.12	−0.13 ± 0.13	−0.01 ± 0.11	−0.08 ± 0.14	0.00 ± 0.11
Irrelevant (*n* = 27)	FP2-FP1	0.05 ± 0.31	−0.05 ± 0.29	0.05 ± 0.26	−0.04 ± 0.29	−0.04 ± 0.24	0.06 ± 0.34
	F4-F3	0.00 ± 0.20	−0.10 ± 0.17	−0.00 ± 0.23	−0.09 ± 0.16	−0.11 ± 0.21	0.00 ± 0.18
	F8-F7	−0.05 ± 0.18	−0.17 ± 0.19	−0.02 ± 0.16	−0.19 ± 0.19	−0.19 ± 0.19	−0.05 ± 0.18
	Frontal R-L	−0.01 ± 0.15	−0.12 ± 0.14	−0.00 ± 0.15	−0.11 ± 0.14	−0.11 ± 0.14	−0.00 ± 0.15
Non-writing (*n* = 25)	FP2-FP1	0.06 ± 0.24	−0.02 ± 0.25	−0.00 ± 0.28	−0.02 ± 0.21	−0.03 ± 0.24	0.08 ± 0.26
	F4-F3	0.02 ± 0.21	−0.07 ± 0.20	−0.07 ± 0.24	−0.07 ± 0.28	−0.08 ± 0.20	0.02 ± 0.21
	F8-F7	−0.05 ± 0.19	−0.13 ± 0.15	−0.13 ± 0.17	−0.13 ± 0.17	−0.15 ± 0.23	−0.05 ± 0.18
	Frontal R-L	−0.01 ± 0.15	−0.09 ± 0.14	−0.08 ± 0.16	−0.08 ± 0.12	−0.09 ± 0.16	−0.00 ± 0.15

**Figure 3:**
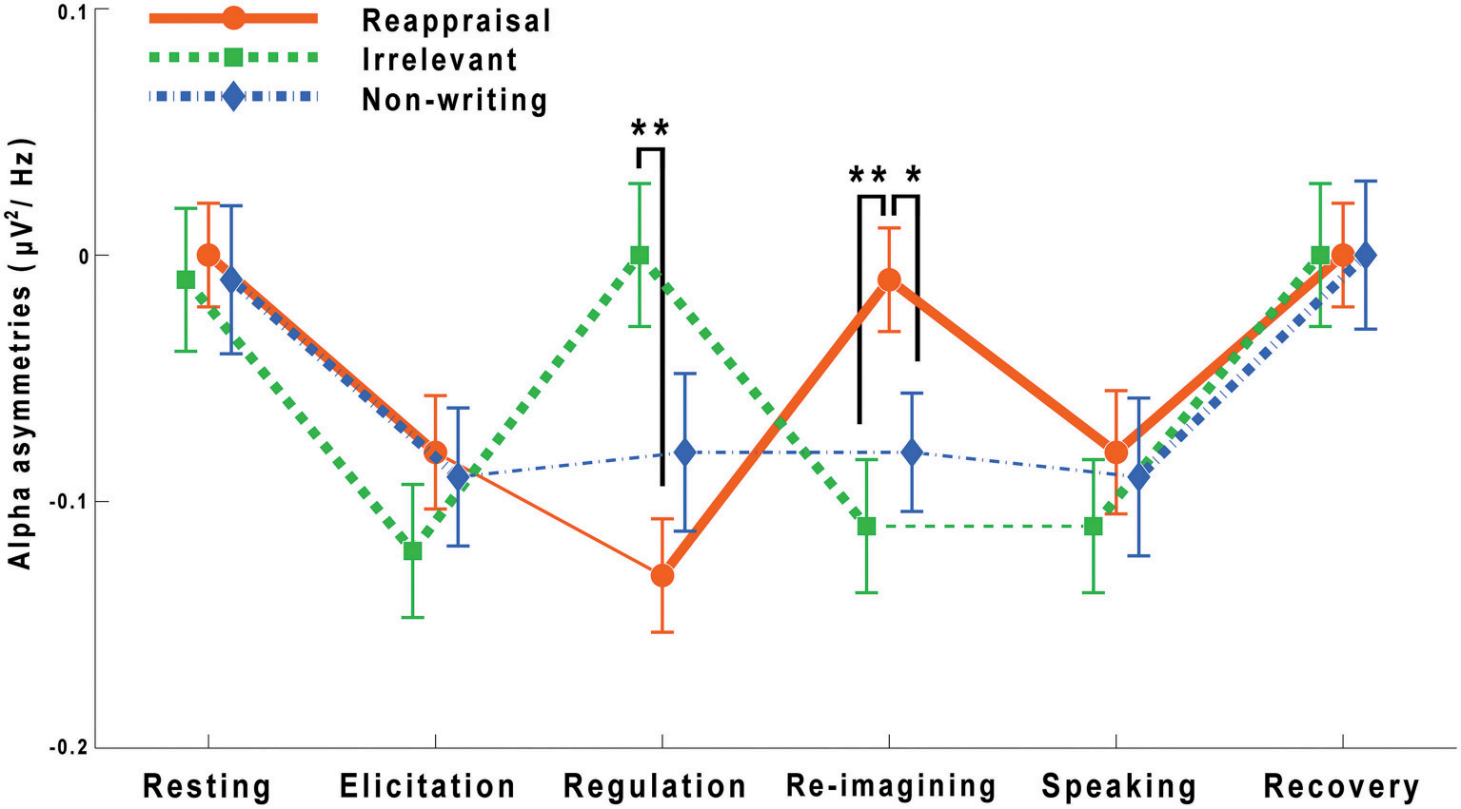
**Frontal alpha asymmetries throughout the entire task**. Averaged FAAs (M ± SEM) of the frontal R-L pair are shown. The asterisks on the top of the figure indicate significant group differences at the regulation stage and the re-imagining stage (^*^P < 0.05, ^**^P < 0.01). The thicker line segments indicate significant differences of adjacent stages for each group.

The following statistical analyses were conducted, based on the averaged frontal asymmetries of the frontal right vs. left pair. FAA changes are also shown in Figure 3.

As with the analysis of the self-reported anxiety scores, a three (group: reappraisal writing, irrelevant writing and non-writing) by six (stage: resting, elicitation, regulation, re-imagining, speaking, and recovery) repeated measures ANOVA was done with the frontal asymmetry scores. There was a significant main effect of stage [*F*_(5, 77)_ = 33.459, *P* < 0.001] and a significant interaction between group and stage [*F*_(10, 77)_ = 9.385, *P* < 0.001]. The group main effect was not significant, *F*_(2, 77)_ = 0.046, *P* > 0.05.

Then six One-way ANOVAs were run to test the group differences for each stage. Groups differed significantly for the regulation [*F*_(2, 77)_ = 5.172, *P* < 0.01] and re-imagining [*F*_(2, 77)_ = 4.855, *P* < 0.05] stages. Multiple comparisons, with Tukey-HSD correction, showed that during the regulation period, participants in the reappraisal group had lower FAA scores than those in the irrelevant writing group (*P* < 0.01). However, during the following re-imagining stage, participants in the reappraisal group showed higher FAA scores than those in the irrelevant writing group (*P* < 0.01). Meanwhile, the multiple comparisons with least significant difference indicated higher FAA scores of the reappraisal group than those of the non-writing group (*P* < 0.05) in the re-imagining stage. There were no other significant findings with regards to the group differences for individual stages.

Three One-way ANOVAs were used to test the stage effects for each group. It was found that for all the three groups, the stage effects were significant: reappraisal writing, *F*_(5, 77)_ = 19.17, *P* < 0.001; irrelevant writing, *F*_(5, 77)_ = 22.80, *P* < 0.001; non-writing, *F*_(5, 77)_ = 10.32, *P* < 0.001. Multiple comparisons with Šidák correction showed that in the reappraisal group, there were significant differences between all adjacent stages (*P* < 0.001 ~ *P* < 0.05) except the elicitation-regulation pair (*P* > 0.05). In the irrelevant group, there was no difference for the re-imagining vs. speaking pair (*P* > 0.05) whereas significant differences were revealed for all other adjacent periods (*P* < 0.001 in all four pairs). In the non-writing condition, there were no significant differences for the elicitation vs. regulation, the regulation vs. re-imagining, or the re-imagining vs. speaking pairs (*P* > 0.05 in all comparisons). In the non-writing group, the elicitation stage decreased FAA significantly from the resting stage (*P* < 0.001), and the recovery increased FAA score significantly from the speaking stage (*P* < 0.01).

### Association between behavioral change and EEG change

Two-tailed Pearson correlation analyses between changes of self-reported anxiety and changes of frontal alpha asymmetry were conducted. Generally speaking, the results (see Table 3) suggested there might be a direction of negative correlation between behavioral changes and EEG changes, i.e., FAA increased when self-reported anxiety levels decreased. But significant correlations were only found when speaking stage minus re-imagining stage for the non-writing group (*P* < 0.05) and across all participants (*P* < 0.01). There was also a significant negative correlation (*P* < 0.05) when recovery minus speaking for the non-writing group. For all participants the negative correlations when regulation minus elicitation (*P* = 0.066) and when recovery minus speaking (*P* = 0.082) reached marginal significance.

**Table 3:** **Bivariate correlations between changes of self-reported anxiety scores and changes of FAA for each group and across all participants**.

**Group**	**Elicitation-Resting**	**Regulation-Elicitation**	**Re-imagining-Regulation**	**Speaking-Re-imagining**	**Recovery-Speaking**
Reappraisal (*n* = 28)	−0.147	−0.04	−0.228	−0.202	−0.053
Irrelevant (*n* = 27)	−0.129	0.082	−0.025	−0.322	−0.231
Non-writing (*n* = 25)	0.023	−0.330	−0.302	−0.458^**^	−0.398^**^
All (*n* = 80)	−0.085	−0.206^*^	−0.185	−0.302^***^	−0.196^*^

* P < 0.1,

** P < 0.05,

*** P < 0.01.

## Discussion

One group of participants in the current study tried to alleviate their speaking anxiety with expressive writing that was instructed to be in a reappraisal style. The regulation effect of reappraisal writing was compared to that of irrelevant writing and a non-writing condition. Self-reported results showed that anxiety levels rose significantly in all groups after anxiety elicitation. Thereafter, anxiety regulation with both reappraisal and irrelevant writing decreased anxiety levels. However, when re-imagining the speaking situation, participants' anxiety increased again in the irrelevant writing condition, while there was no change in the reappraisal condition. From re-imagining to speaking, anxiety rose in all groups. In addition, in the recovery stage, all groups recovered to the same anxiety level. As for the EEG FAA, anxiety elicitation reduced FAA, compared to the resting stage, in the three groups. Regulation with reappraisal did not change FAA levels, but the irrelevant writing led to an increase of FAA. Interestingly, in the re-imagining stage, compared to the regulation stage, reappraisal writing increased FAA but irrelevant writing lowered FAA. The FAA level in the reappraisal group dropped again from re-imagining to the speaking stage, and there was no group difference in the speaking period. Finally, the recovery stage made FAA rise to the same level in all groups.

These results indicate that expressive writing can attenuate negative emotions to some degree, and reappraisal may have an important role in this process. The current finding is consistent with many previous studies on emotion regulation (e.g., Hajcak and Nieuwenhuis, 2006; Goldin et al., 2008; Hofmann et al., 2009; Andreotti et al., 2013) in which reappraisal was conducted by thinking. By re-evaluating an emotional event's meaning in other ways, reappraisal can successfully change the subjective experience. Writing reappraisal in the present study achieved the same effect.

The neural correlates of reappraisal thinking have been reported by some previous studies. For example, reappraisal can result in a decrease in the amplitudes of the late positive potential (LPP) (Hajcak and Nieuwenhuis, 2006). The prefrontal cortex may play an important role in the reappraisal processes (e.g., Goldin et al., 2008; Winecoff et al., 2013). However, what are the neural electrical changes related to reappraisal writing? From the change directions of group averages, this study found that FAA corresponded to self-reported anxiety in general. By and large, reappraisal writing had a similar effect when measured by self-report and FAA. According to Davidson's approach-withdrawal theory (Davidson, 1990, 1992, 1998) that tries to explain the association between EEG asymmetry and affective behaviors, relatively greater left-sided activation is associated with approach tendencies toward emotional stimuli, while relatively right-sided activation is related to tendencies to withdraw from stimuli. A growing literature has also indicated that frontal EEG asymmetry may act as a risk factor for a variety of emotion-related disorders, including depression and anxiety (Coan and Allen, 2004). Although, there are some inconsistencies in the findings, the link between resting frontal EEG asymmetry and depression and anxiety has been supported by meta-analysis (Thibodeau et al., 2006). Many studies have paid attention to the relationship between resting EEG and psychopathology. Most of them reported increased right-sided resting frontal asymmetry in depressed and anxious individuals, including those with social anxiety (e.g., Petruzzello and Landers, 1994; Debener et al., 2000; Allen et al., 2004; Moscovitch et al., 2011). In addition to resting EEG, it is reported that frontal EEG asymmetry acquired in the task stage is also linked to an anxious state (e.g., Davidson et al., 2000). For example, during speech preparation, some individuals showed increases in right FAA from baseline to the stressful speech condition, and the increased frontal asymmetry was associated with vigilance to angry faces and avoidance of happy faces (Pérez-Edgar et al., 2013). Under highly stressful conditions, participants with a higher state asymmetry score exhibited greater emotion regulation as indexed by significant attenuation of eyeblink startle magnitudes (Goodman et al., 2013).

In line with previous findings (e.g., Davidson et al., 2000; Moscovitch et al., 2011; Pérez-Edgar et al., 2013), the current study observed an increase of FAA, along with a decrease in self-reported anxiety in general. Of interest, the relationship between subjective anxiety and FAA did not always comply with the pattern described above. From the elicitation stage to the regulation stage, although self-reported anxiety decreased significantly, reappraisal writing did not lead to a significant change in FAA. One possibility is that when reappraisal writing pushed participants to face and re-evaluate the stressful situation, in a relatively short period participants had to endure some extent of anxiety because they had been exposed to the stressful event and were required to think of it. The anxiety-like pattern of frontal EEG asymmetry during reappraisal writing may be the manifestation of this exposure effect. As for the self-reported attenuation of anxiety, because the data were collected after the completion of regulation, participants might have passed the temporary anxious exposure and gained the benefits of regulation. Of course, there may be some other possible explanations. For example, participants' inference of the experimental aim, and associated ingratiation behavior, may contribute to the subjective report results. The suggestion effect from the experimental instructions may be another reason. Anyway, it is worthwhile to note that timing may be an important factor for studying the effect of expressive writing. Further, evidence can be obtained when combining elicitation, regulation and re-imagining stages together. From regulation to re-imagining, expressive writing did not make a change in subjective anxiety, while the FAA increased significantly. Thereafter, from a pretest-posttest angle, it can be concluded that expressive writing decreased anxiety about public speaking. A FAA increase followed the decrease of self-reported anxiety, but it might not occur very quickly. In addition, a relatively slow-acting effect may be one of the characteristics of expressive writing, because writing is not as fast as thinking.

It is noteworthy that although the current study found some degree of correspondence between subjective feeling changes and the fluctuation of FAA, the association was not strong according to the current findings. There was a coarse corresponding relationship between behavioral changes and EEG changes in the group average level. However, correlation analyses could reach significance in only a few places. Large individual differences existed in the current study. Based on the present results it is difficult to make a precise and robust inference about the relationship between anxiety changes and FAA changes. In addition, in the studies of Debener et al. (2000) and Allen et al. (2004), they did not find that changes in resting frontal asymmetry were related to patients' clinical status or mood. Due to the significant differences in participants, tasks, and observation indexes, it is hard to make further judgment based on existing reports. More work is still needed to explore this issue. In any case, caution should be exercised regarding the current findings.

Another interesting finding in this study is that irrelevant writing seems to have reduced anxiety in the regulation stage. However, in most other studies on expressive writing (e.g., Gortner et al., 2006; Ramirez and Beilock, 2011), irrelevant writing usually has no regulation effect. Are the results of this study at odds with those reported previously? After the experiment, some participants mentioned that “writing my weekly plan made me forget the speech task for a moment,” but “thinking about the speech task made me nervous.” We propose that the irrelevant writing may act as a distractor. Indeed, the current manipulation of irrelevant writing fits the concept of distraction very well. When people use distraction to regulate emotions, they may divert attention from the emotional aspects of the stimulus or even replace the current attentional focus with totally irrelevant thoughts or memories (Gross, 2014). It is widely reported that distraction can reduce subjective emotional feelings and bring about corresponding neural changes (e.g., Kanske et al., 2011; Lieberman et al., 2011; Schönfelder et al., 2013; Simon et al., 2014). In the present study, writing a weekly plan successfully moved participants' attention from the stressful public speaking task and thus gave them a temporary mental respite from the stressor. Therefore, it is understandable to find decreases in subjective anxiety and increases in FAA from elicitation to regulation. It should be noted that the anxiety-easing effect of irrelevant writing did not persist. When participants imagined the speech task again, their anxiety levels increased significantly along with decreased FAA. There is one ERP study (Thiruchselvam et al., 2011) echoing the current result to some extent. It found that when participants diverted attention away from affective pictures, their LPP amplitudes decreased significantly. However, when participants were exposed to these pictures again, they elicited larger LPP amplitudes than those with an attendant history. This study indicated that distraction can attenuate emotional responses in the short term but that this effect may come at a cost by eliciting larger emotional responses in the long run. Thus, it is not hard to understand why irrelevant writing could alleviate anxiety in the regulation stage but this effect disappeared when participants were exposed to the anxiety-eliciting situation again. In previous studies, the efficacy of expressive writing is usually evidenced by tests that are conducted sometime after the completion of writing. At that time, the temporary regulation effect of irrelevant writing may have decreased dramatically. Although distraction can attenuate emotion rapidly, it is not recommended in the process of expressive writing. Avoiding emotional stimulation may play a role of mental protection in the acute stress stage, but may also result in losing the opportunity to face and handle the current emotional event. In the long run, it may therefore cause maladaptive consequences, especially when some events become inescapable. Expressive writing emphasizes the confrontation with and disclosure of one's own emotions, exposure to the emotion-eliciting situations, and exploring possible cognitive changes. These approaches lead to a more thorough resolution of mental problems.

Some may have expected an alleviation of anxiety in the speaking stage after the reappraisal writing, but the present results showed that, as in the irrelevant writing and the non-writing groups, self-reported anxiety in the reappraisal group also increased from the re-imagining stage to the speaking stage. Meanwhile, although the FAA score of the reappraisal group increased from the regulation to the re-imagining stage, public speaking decreased it to a similar level as that in the irrelevant writing and the non-writing groups. This indicates that in the current experimental paradigm, although reappraisal writing can exert some effects of anxiety relief, these effects are relatively weak compared to those elicited by stressful speaking task. A short-term regulation is not enough to overcome the powerful influence of speaking anxiety. Additionally, perhaps just as Pennebaker and Chung (2011) stated, expressive writing takes effect through multiple mechanisms, whereas in the current study participants were mainly instructed to make cognitive changes related to the public speaking task. This restriction may have reduced the regulation effect to some degree. Therefore, we can see the importance of systematic intervention when trying to resolve mental problems. An adequate course of treatment, deep disclosure and thorough cognitive change, and synthetic application of multiple interventions or therapeutics may be helpful to improve its curative effect.

In conclusion, the present study found that expressive writing in a reappraisal style could decrease speaking anxiety to some extent. This effect was accompanied by a decrease in right frontal cortical activation. Facing the anxiety-eliciting event and reappraising the situation may be a useful approach to relieve anxiety. However, in the current study the association between subjective feeling and EEG activity existed only in the change direction of group means and a few of correlation analyses. The precise relationship still cannot be elucidated clearly by the current findings. Meanwhile, the individual differences may have been submerged in the group averages. It should be cautious when using the current results to consider the problem of particular individuals. In addition, the present study suggests that the acting time of expressive writing deserves more attention. Further work is still needed to examine the mechanisms of expressive writing and to improve its practical applications.

## Author contributions

FW, YH, CW, and CZ designed the experiment. FW, DL, and MM performed the experiments. YH, CW, FW, QY, and KW analyzed the data. All authors discussed the results and contributed to the writing of the paper.

### Conflict of interest statement

The authors declare that the research was conducted in the absence of any commercial or financial relationships that could be construed as a potential conflict of interest.
