# Supplementary material for: Reappraisal writing relieves social anxiety and may be accompanied by changes in frontal alpha asymmetry
Source: Front Psychol. 2015 Oct 21;6:1604. doi: 10.3389/fpsyg.2015.01604 (PMC4612649; doi:10.3389/fpsyg.2015.01604)
Supplement: Supplementary file 1 [file DataSheet1.DOCX]

**Instructions:**

In the anxiety elicitation stage, the participants were given the following instructions (in Chinese): *At the end of the experiment, you will be asked to give a 3-minute impromptu speech in English. Your performance will be evaluated by three experimenters at that moment and will be videotaped for further analysis. The topic of the speech will be presented 1 minute before your speech. The requirements of the speech include: a) keep to the point and be informative; b) express yourself clearly and fluently; c) keep strict logic and coherence.* After the participants had finished reading the instructions above, they were presented with the following instructions: *Please imagine the speech scenario as vividly as possible, such as how difficult the speech topic will be, your own performance and the evaluation from the experimenters, or recall an embarrassing situation you have encountered in a past speech experience. The time for imagining or recalling is 2 minutes, and please keep your eyes open.*

In the regulation stage, the instructions for the reappraisal writing group were: *Before the speech, please adjust your emotions using the following tips and write down all of the processes. You can think that you will benefit from the speech, such as with a payment or through speech experience; you can think that the speech is just an experiment so it is no big deal no matter what happens; you can think that being nervous is quite normal and maybe some level of anxiety will inspire better performance. You need not pay attention to your handwriting. The time for this procedure is 5 minutes.* The instructions for the irrelevant writing group were as follows: *Before the speech, please adjust your emotions using the following tips and write down all of the processes. You can think of your weekly plan for the next week in detail. You need not pay attention to your handwriting. The time for this procedure is 5 minutes.* The instructions for the non-writing group were as follows: *Before the speech, we need to record your EEG signal for further analysis. Please sit still and keep your eyes open. The time for this procedure is 5 minutes.*

In the speaking stage, the participants were given the following written instructions before the speech: *The topic of your speech is “Can knowledge change one’s fate?” You have 1 minute to prepare. Your speech must be in English, and the time for your speech is 3 minutes.*
